# How I Built My Personal, Patient-Centered Health Care Team: Instead of Doctors, I Started With Students

**DOI:** 10.2196/44693

**Published:** 2023-02-06

**Authors:** Richard Wassersug

**Affiliations:** 1 University of British Columbia North Vancouver, BC Canada

**Keywords:** prostate cancer, mentorship, medical education, students, patient with cancer, urologist, support, researchers, patient-centered, colleagues, health care training

## Abstract

As a patient with cancer, I witnessed how beneficial it was to be treated by a multidisciplinary health care team. I realized I already had my own team, in a sense. That is because I had treated my research students as colleagues from the get-go, and I did not abandon them when they graduated and moved on.

Access to health care is challenging these days. Wait times to see a doctor in a walk-in clinic or emergency room have become inordinately protracted. For anyone not in critical condition, but still not feeling well, waiting for health care can become an activity of daily living. Even if you are lucky enough to have a general practitioner and are a Doctor of Medicine yourself, getting a timely appointment is not easy.

By the time I was in my mid-50s, I knew a day would come when I would need a health care team I could access quickly and trust explicitly. I hoped—whatever medical problem might emerge—I would have a team in place well in advance of needing their professional services.

What brought this to mind was getting diagnosed with prostate cancer when I was in my early 50s. In relatively short order, I was treated by a urologist, a radiation oncologist, and a medical oncologist. I experienced firsthand how comprehensive cancer care requires many specialists upfront and more backing them up. For instance, a pathologist confirmed my diagnosis based on biopsy samples collected by a urologist. The urologist, who removed my prostate gland, had another surgeon assisting him in the operating room. An anesthesiologist in the same room made sure that I got out of there with no functional losses other than those that went with the cancerous organ the surgeon removed.

Despite having witnessed how beneficial it was as a patient with cancer to be treated by a multidisciplinary health care team, I did not immediately set out to build one. In retrospect, however, that is what I managed to do, and I feel lucky that I did—for blood markers suggest that my cancer will eventually return.

So here is how I built that team, and how others can do it.

Though I lack any training in health care, I was a university science professor with a lot of contact with undergraduates. So that is what I had to work with, and that is where my team-building program began.

I did not particularly care if the students taking my classes wanted to be medical specialists or doctors of any ilk. Many of them were still teenagers, too early in their training to make that call; but I needed research collaborators; so, whenever I came across brilliant, enthusiastic, curious students with solid A transcripts, I made them an offer. The offer was a chance to do original research with me worthy of publication. As luck would have it, many accepted the offer.

Since the students were overall superb, I was genuinely happy to support them in whatever professional pursuits caught their interest. Many chose postbachelor training in health care, which demands a passion for problem-solving. That fit well with my research.

Over the years, I learned from the students about the intricacies of health care training in a variety of fields. Increasingly, I found myself encouraging my student collaborators toward careers in health care. Indeed, the same traits of brilliance, enthusiasm, and curiosity that make for good scientists also make for good health care providers.

“What,” you may ask, “was the outcome?” Here is a summary, without naming specific students.

Let us start with the general practitioners, since health care typically starts with a family doctor. There are currently 5 licensed general practitioners who started working with me long before they went to medical school. One ex-student is now in internal medicine with specialist training as an intensivist. That is especially good because I might end up at some point in the intensive care unit. Two are oncologists. I consider that a big win. One, who is still in medical school, is heading for surgery, and another is a resident in anesthesiology. Thus, the surgeon will have a wing man in the operating room. Another is finishing her training as a pulmonologist, which is great, as I have now got some pulmonary issues that need monitoring.

That is pretty good coverage in terms of the medical specialists I may need. But my personal health care team is not complete.

Another undergraduate collaborator, who is still in medical school, tells me she likes her rotation in gerontology (yes!), but also says she likes pediatrics (sigh). I cannot win them all and have already had to accept some failures. For example, one of my ex-undergraduates is a resident in pediatrics, and another is a board-certified obstetrician. Those specialties are no use to me, but I know those women well enough to know that many others will benefit from their diligence and commitment to excellence. It was indeed a joy collaborating with them for they are natural researchers (and remain close friends).

I like to believe I am sane, but I realize that if my heart, lungs, and kidneys can fail, so can my brain. Thus, it is nice to know that one ex-student co-author is now a resident in psychiatry, and another is a psychiatric nurse. Broadening my coverage, 3 ex-students who have published extensively with me are now board-certified PhD clinical psychologists.

We do not live forever, and life will get rough when the asymptomatic tumors I have start to grow. In that regard, I am pleased that one of the general practitioners has specialist training in palliative care. I cannot avoid dying, but I am glad to have someone with expertise in managing pain whom I might confer with if my cancer reemerges.

What all these professionals have in common is that they started their training as researchers long before they became health care providers. Collectively we tackled a slew of fun projects in a wide variety of fields. Few of our projects had anything to do with health care, but that did not matter. The students were willing to take on whatever weird project I thought worth investigating. I, in turn, was willing to back them in pursuing whatever career caught their eye.

Back then, I treated my research students as colleagues from the get-go and not as transient laborers just passing through the lab. I also did not abandon them when they graduated and moved on.

Admittedly, over the decades, many of these colleagues drifted away from the university where we first met, and they are now dispersed across 6 Canadian provinces and 3 countries. Online communication has kept them, my health care team, in my view.

I realize, in retrospect, that I constructed a personal, patient-centered health care team. I cannot claim that my team-building enterprise was perfect. Modern medicine is vastly complex—hence the need for not just individual clinicians but health care teams—and there were limits on how many students I could manage at any one time. However, none of the students I worked with are to me distant and detached intimidating folks in white coats. They are real friends and colleagues *from way back when*.

The implicit social contract was that I never abandoned my students regardless of their evolving interests and pursuits, and I do not anticipate that they will abandon me.

I have never liked the lopsided egotism when patients cry out for more *patient-centered care* as they complain about health care providers who they felt did not give them enough time and attention. What is too often missed from these demands is much understanding that our health care providers are people too. In reciprocal fashion, if we want health care providers to care about us, we should care about them. I made my personal, patient-centered health care team by first running a student-centered program. Core to that exercise was treating students as colleagues, not underlings.

## You Do Not Need a Research Laboratory

Good health care starts with truly caring for others. Health care professionals are humans just like us, who need care at all stages of their lives. Getting good health care providers, who work as a team on our behalf, can start with us demonstrably caring for them even before they are qualified to care for us.

Sure, I want to be the center of attention if and when I go back to being a patient with cancer, in need of intensive care. But in reciprocal fashion, we all can preemptively care for the health care providers of the future long before they start their professional training. This requires recognizing brilliant, enthusiastic, curious youth and endorsing their commitment to excellence however it is manifested.

What I have learned from life as both an educator and patient with cancer is that getting patient-centered care can be accomplished in the long run by starting with student-centered care. The key was building personal, equalitarian relationships with students long before they had locked into training as health care providers.

Over the years, I have met Doctors of Medicine with undergraduate degrees in a vast array of fields, including English literature, music, and philosophy. Most did not begin university committed to health care, but they were committed to excellence.

In that regard, educators in almost any field can build a health care team simply by inviting their very best students to be their collaborators. Those students do not need to be initially committed to health care, but they do need to be committed to excellence in whatever they do… and we should be committed to them as early as possible.

I am confident that these health care providers, who I worked with early in their schooling, will be caring professionals when I need them.

